# Study on the Fingerprint Spectrum and the Spectrum-Effect Relationship of Analgesic and Anti-Inflammatory Effects of the Aqueous Extract from *Dalbergia hancai* Benth

**DOI:** 10.1155/2023/1242756

**Published:** 2023-06-23

**Authors:** Qin Qiu, Xiaofang Liu, Chunying Huang, Yanling Guo, Dandan Zhen, Junhao Shi, Baojun Gu, Hanshen Zhen, Miao Zhang

**Affiliations:** ^1^Guangxi University of Chinese Medical, Nanning 530200, China; ^2^Traditional Chinese Medicine Hospital of YuLin, Yulin 537099, China; ^3^The People's Hospital of WuZhou, Wuzhou 543000, China

## Abstract

*Dalbergia hancai* Benth. (*D. hancai*) is one of the most frequently utilized traditional Chinese medicine in Zhuang medicine. Simultaneously, it has been included in the “Quality Standard of Zhuang medicine in Guangxi Zhuang Autonomous Region (Vol. 2)” and possessed outstanding pharmacological effects. However, the pharmacodynamic material basis of *D. hancai* still remains unclear. In this study, the high-performance liquid chromatography (HPLC) method had been employed to establish the fingerprint of 10 batches of aqueous extract of *D. hancai* originated from different parts of China. At the same time, similarity evaluation, cluster analysis, and principal component analysis (PCA) had also been conducted to evaluate the common peaks. The acetic acid-induced writhing in mice had been employed as an analgesic model, and the carrageenan-induced toe swelling in mice was utilized as an anti-inflammatory model for pharmacodynamic experiments. The gray relational analysis (GRA) and partial least squares regression (PLSR) were applied to correlate the fingerprint and pharmacodynamic data to thoroughly examine its spectrum-effect relationship, whereby its analgesic and anti-inflammatory material basis had been comprehensively explored. The results revealed that the HPLC fingerprint of the aqueous extract of *D. hancai* had successfully identified 12 common peaks whereby two of which were further identified as protocatechuic acid and vitexin. Subsequently, through the analysis of GRA and PLSR, the chromatographic peaks that possess a critical correlation degree with the analgesic and anti-inflammatory effects of *D. hancai* had also been successfully discovered. Ultimately, the analgesic and anti-inflammatory effects of the 10 batches of *D. hancai* aqueous extract had been conclusively proved, and it was evidently indicated that these effects were attributable to the synergistic interactions between various components. Therefore, this study aims to serve as an effective analytical method for screening and predicting the effective substances of traditional Chinese medicine on the basis of the spectrum-effect relationship.

## 1. Introduction

*Dalbergia hancei* Benth (*D. hancai*), a Zhuang medicine, is a plant of *Dalbergia* (*Fabaceae*), which is predominantly distributed in Guangxi, Guangdong, Guizhou, and various other places in China. The roots and stems have been utilized as the medicinal parts of *D. hancai* [1]. As one of the commonly utilized folk prescriptions of the Guangxi Zhuang nationality, it possesses a long history of application and has demonstrated the effects of regulating qi, relieving pain, tendon-relaxing, and collateral-activating. At the same time, it has also been employed to treat lumbago, arthralgia, abdominal pain, and so on [2, 3]. Modern pharmacological studies have demonstrated that *Dalbergia L. f*. plants possess excellent analgesic and anti-inflammatory effects [4, 5]. *D. hancai* is a plant of *Dalbergia L. f*. At present, there are relatively minimal reports that focused on its chemical constituents and pharmacological effects, as well as providing a quality analysis of *D. hancai* [6]. The existing research only revealed a preliminary understanding of the chemical constituents such as tannins, flavonoids, sterols, and triterpenoids in the stem bark of *D. hancai* [7]. As such, it is immensely challenging to accurately and effectively evaluate the quality of *D. hancai*. Hence, it is extremely vital to comprehensively investigate the quality of *D. hancai* from various different origins, so as to provide a certain reference for its development, utilization, and clinical use.

Nowadays, due to the absence of accurate and effective quality analysis as well as quality control methods, numerous traditional Chinese medicine which possess outstanding medicinal value, have not been fully developed and utilized. The chromatogram or spectrum of the common peaks acquired by a specific modern analysis technology following the correct treatment of Chinese herbal medicine or Chinese medicine preparation, which can clearly reflect the chemical information of the plant, is referred to as the “fingerprint” of traditional Chinese medicine. This spectrum will be able to clearly reveal the population characteristics of each component and therefore able to accurately reflect the population characteristics of the plant. However, due to the unavoidable complexity of the components that existed in traditional Chinese medicine and that its efficacy is the result of the coordination and comprehensive action of various components [8–10]. The use of fingerprint cannot directly evaluate the efficacy of drugs, and only the chemical information obtained by fingerprint to standardize and control the quality of traditional Chinese medicine is not comprehensive. Thus, the safety and efficacy of drugs should also be selected as the relevant indicators of quality control. Simultaneously, performing pharmacodynamic studies to integrate the quality and efficacy of Chinese medicines can facilitate the clarification of the mechanism of effect [11]. The spectrum-effect theory of traditional Chinese medicine will be able to effectively resolve the above problems. By associating traditional Chinese medicine's fingerprint with pharmacodynamics through chemometrics models, it signifies the development of a thorough assessment method incorporating chemical analysis and biological activity evaluation under the guideline of traditional Chinese medicine theory in order to effectively uncover the physical underpinnings of traditional Chinese medicine's effectiveness [12]. On the one hand, the “spectrum-effect” research method will be able to make up for the shortcomings of the traditional Chinese medicine research model that focuses on component research and ignores pharmacodynamic research. On the other hand, it is able to effectively realize the organic combination of traditional Chinese medicine fingerprint and pharmacodynamic research, as well as supplement the necessary pharmacodynamic information of traditional Chinese medicine to the “spectrum” so as to successfully achieve the purpose of accurately predicting the efficacy of traditional Chinese medicine according to the “spectrum” and enhancing the consistency of “spectrum” and “effect” [13]. At present, the spectrum-effect relationship has been widely adopted in the study of the relationship between active components and the pharmacological effects of traditional Chinese medicine [14–16].

In our previous research, a hot plate test, acetic acid writhing test, xylene-induced ear swelling test in mice, and carrageenan-induced toe swelling test in mice had been employed to thoroughly investigate the analgesic and anti-inflammatory effects of high, medium, and low doses of aqueous extract of *D. hancai*. It was discovered that the high and medium doses of aqueous extract of *D. hancai* possessed excellent analgesic and anti-inflammatory effects. Therefore, in order to further evaluate the analgesic and anti-inflammatory active substances of *D. hancai*, this experiment aims to establish the high-performance liquid chromatography (HPLC) fingerprint of *D. hancai* aqueous extract so as to comprehensively investigate the analgesic and anti-inflammatory effects of *D. hancai* aqueous extract from different origins. The data of fingerprint and pharmacodynamic indexes are correlated by gray relational analysis (GRA) and partial least squares regression (PLSR). The relationship between the “spectrum” and “effect” of *D. hancai* had been analyzed, and the pharmacodynamic components related to analgesic and anti-inflammatory effects were screened out, respectively. Subsequently, the pharmacodynamic basis of analgesic and anti-inflammatory effects of *D. hancai* was preliminarily discussed in order to serve as an experimental basis for further exploration of analgesic and anti-inflammatory pharmacodynamic substances, quality control research, and further development along with the utilization of *D. hancai*.

## 2. Materials and Methods

### 2.1. Samples, Reagents, and Animals

The 10 batches of *D. hancai* from different origins were collected from Guangxi province. The plants were identified as the stems of *D. hancai* belonging to *Dalbergia* (Fabaceae) by Ma Lifei, Deputy Chief Pharmacist (Deputy Chief Pharmacist of Guangxi Yixin Pharmaceutical Co., Ltd.). See Table 1 for details. The protocatechuic acid reference substance was purchased from China Institute for the Control of Pharmaceutical and Biological Products (Beijing, China, No: 110809-200503); Vitexin reference substance was purchased from China Institute for the Control of Pharmaceutical and Biological Products (Beijing, China, No: 111687-201704); Rotundine tablets Sichuan were purchased from Difit Pharmaceutical Co., Ltd. (Sichuan, China, No: 200601); Glacial acetic acid were purchased from Tianjin Fuyu Fine Chemical Co., Ltd. (Tianjin, China, No: 20200722); Dexamethasone acetate tablets were purchased from Zhejiang Xianju Pharmaceutical Co., Ltd. (Zhejiang, China, No: 200305); carrageenan for reagent grade, were purchased from Shanghai McLin Biochemical Technology Co., Ltd. (Shanghai, China, No: C10207866); Acetonitrile and methanol are chromatographic pure, other reagent is analytical pure, water is ultrapure water.

KM mice (SPF grade), male, weighing 18∼22 g, were supplied by Hunan Slake Jingda Laboratory Animal Co., Ltd. (approval no: SCXK (Xiang) 2019-0004. Changsha, Hunan, China). Mice were fed at room temperature (22°C ± 2°C) for 12 hours under a light/dark cycle. The Animal Ethics Committee of the Guangxi University of Chinese Medicine approved all animal protocols. (approval no. DW20201025-136). The animal experiments were carried out according to the Guide for the Care and Use of Laboratory Animals.

### 2.2. Apparatus and Conditions

The reformulate results are as follows: LC-20AT high performance liquid chromatograph, SPD-M20A detector (Shimadzu Corporation, Japan). Waters Atlantis C18 column (4.60 mm × 250 mm, 5 *μ*m); column temperature, 35°C; detection wavelength, 260 nm; the flow rate was 0.8 mL·min-1; the injection volume was 20 *μ*L; the mobile phase was a mixture of methanol (A) and 0.1% phosphoric acid solution (B). The gradient elution: 0-7 min, 5%-10% A; 7-15 min, 10%-15% A; 15-20 min, 15% A; 20-40 min, 15%-25% A; 40-80 min, 25%-27% A; 80-98 min, 27%-38% A.

### 2.3. Preparation of Samples Solution

#### 2.3.1. Preparation of Sample Solution for HPLC Fingerprint Analysis

2 g of *D. hancai* powder was extracted with 25 mL of distilled water in a plugged conical flask for 1 h. Subsequently, it was cooled to room temperature and distilled water was added to make up the original weight before being shaken and filtered. The filtrate was centrifuged at a speed of 13 000 r·min^−1^ for 10 min, and the supernatant was utilized as the test solution for HPLC analysis.

#### 2.3.2. Preparation of Reference Solution

Appropriate amounts of protocatechuic acid and vitexin reference substance were precisely weighed, and methanol was used to prepare protocatechuic acid reference substance storage solution (0.0512 mg·mL^−1^) and vitexin reference substance storage solution (0.0485 mg·mL^−1^), respectively.

#### 2.3.3. Samples Solution for Animal Experiments Administration

The fresh medicinal materials of *D. hancai* were dried in an oven at 60°C and crushed into powder. The powder of *D. hancai* was repeatedly extracted with distilled water for 3 times. The first extraction was soaked in 10 times the amount of distilled water for 30 min before being heated and refluxed for 1 h. The second and third extractions were directly refluxed with 8 times the amount of distilled water for 1 h. After each extraction, the extract was filtered and the filtrate concentrated with a rotary evaporator. Finally, an aqueous solution was acquired and stored in a refrigerator at 4°C. A total of 10 batches of *D. hancai* medicinal materials from different origins were prepared into extracts from the corresponding producing areas, according to the above methods. When the extract was utilized, distilled water was added to prepare a solution equivalent to 57.6 g/kg of crude drug as a sample solution for animal experiments.

### 2.4. Method Validation of the HPLC Fingerprint

The HPLC fingerprint analysis method had been employed to effectively evaluate the precision, repeatability, and along with the stability of the sample by calculating the relative retention time of each common peak and the RSD value of the relative peak area with the peak 2 protocatechuic acid as the reference peak (S). Under the chromatographic conditions of paragraph “2.2”, the precision was evaluated by continuous injection for 6 times, the repeatability was evaluated by parallel preparation of 6 sample solutions, and lastly the stability was evaluated by measuring and analyzing the samples at 0 h, 2 h, 4 h, 8 h, 12 h, and 24 h.

### 2.5. Animal Experiments

#### 2.5.1. Study on Analgesic Effect

The KM male mice were randomly divided into twelve groups with ten mice in each. Group 1 acted as the model group (mice were given distilled water at the same volume), group 2 was the positive group (mice were given 0.03 g/kg rotundine solution at the same volume) while 3–12 were the medication administration groups (mice were given 57.6 g/kg aqueous extract of *D. hancai* from different origins (S1-S10)). After all the animals had experienced 3 days of the adaptation period, each group had been admitted with the corresponding liquid medicine, and the dosage volume of administration was 0.2 mL/10 g once a day for 7 days. Subsequently, one hour after the last administration, the mice in each individual group were intraperitoneally injected with 0.6% glacial acetic acid solution (injection volume of 0.1 mL/10 g). The number of writhing in mice was observed within 15 minutes (with intraperitoneal depression, hip elevation, and hind limb extension as writhing criteria), and the corresponding analgesic inhibition rate was calculated. The calculation formula is as follows:

Inhibition rate of analgesia (%) = (average writhing times of model group − average writhing times in administration group)/average writhing times of model group × 100%.

#### 2.5.2. Study on Anti-Inflammatory Effect

The KM male mice were randomly divided into twelve groups with eight mice in each. Group 1 acted as the model group (mice were given distilled water at the same volume), group 2 served as the positive group (mice were given 0.006 g/kg dexamethasone acetate solution at the same volume), while groups 3–12 were the medication administration groups (mice were given 57.6 g/kg aqueous extract of *D. hancai* from different origins (S1–S10)). After the animals had experienced 3 days of the adaptation period, each group had been admitted with the corresponding liquid medicine, and the dosage volume of administration was 0.2 mL/10 g once a day for 7 days. One hour after the last administration, 40 *μ*L 1% carrageenan was subcutaneously injected into the right hind toe of mice to induce inflammation, and the left toe was treated as a reference. 30 min later, mice were cervically executed, and both feet were removed from the same part of the ankle joint, precisely weighed, and the difference in weight between the right and left hind feet of the same mouse was used to represent the swelling of the toe, and the swelling inhibition rate was successfully calculated. The calculation formula is as follows: degree of toe swelling = right toe weight − left toe weight; swelling inhibition rate = (the swelling degree of the model group − the swelling degree of the administration group)/the swelling degree of the model group × 100%.

### 2.6. Data Handling

The fingerprint data of 10 batches of aqueous extracts of *D. hancai* from different origins were thoroughly evaluated for similarity by adopting the “Chinese traditional medicine chromatographic fingerprint similarity evaluation system (2012,1 Edition).” The control fingerprint was generated by averaging the reference spectrum of sample S1 with a time window width of 0.1. After multipoint correction, the Mark peaks were automatically aligned to create a control fingerprint(R) with the median method. Subsequently, through the IBM SPSS Statistics 22.0 software, the 12 common peak areas of 10 batches of aqueous extract samples of *D. hancai* from different origins were systematically clustered by employing the method of between groups linkage, and the similarity of the samples was calculated by utilizing the square Euclidean distance as the measurement standard. Concurrently, the IBM SPSS Statistics 22.0 software had also been employed for PCA to effectively calculate the eigenvalue and variance contribution rate.

The IBM SPSS Statistics 22.0 software was utilized for the statistical analysis of animal experimental data. The experimental data were expressed as $\bar{x}\pm s$. At the same time, an independent sample *t*-test was used to effectively compare the differences between the administration group and the model group, and the result of *P* <  0.05 indicated that the difference had been statistically significant.

The 12 common peak areas of the fingerprints of 10 batches of aqueous extract samples of *D. hancai* from different origins were determined as subsequence (*X*). The inhibition rate of the aqueous extract of *D. hancai* on analgesia in mice and the inhibition rate of the swelling in mice were determined as parent sequence (*Y*), respectively. The calculation method and steps were analyzed by gray correlation degree according to references [17–20]. The peak area of each common peak in the fingerprints of 10 batches of aqueous extract of *D. hancai* was utilized as the independent variable *X*, and the inhibition rate of analgesic effect and toe swelling of aqueous extract of *D. hancai* in mice were taken as the dependent variable *Y*, respectively. Lastly, the SIMCA14.0 software was utilized for the PLSR analysis of the analgesic and anti-inflammatory spectrum-effect relationship, respectively.

## 3. Results

### 3.1. HPLC Fingerprint Analysis Results

#### 3.1.1. Method Validation

The results of the precision test showed that the RSD value of the relative retention time of common peaks was 0.05%∼0.17%, and the RSD value of relative peak area was 1.26%∼2.73% (*n* = 6), indicating that the precision of the instrument was good. The repeatability test results showed that the RSD values of the relative retention time of the common peaks were 0.03%∼0.51%, and the RSD values of the relative peak area were 1.32%∼2.99% (*n* = 6), indicating that the method had good repeatability. The results of the stability test showed that the RSD value of the relative retention time of common peaks was 0.08%∼0.29%, and the RSD value of the relative peak area was 0.2%∼2.76%, indicating that the test solution had good stability within 24 h.

#### 3.1.2. HPLC Fingerprint

The HPLC fingerprints of 10 batches of aqueous extracts of *D. hancai* from different origins had been successfully established, and 12 common peaks were determined. Through the reference substance comparison method, peak 2 was successfully identified as protocatechuic acid, and peak 11 was identified as vitexin. The standard substance utilized for identification, inspection, and content determination is referred to as the reference substance. Peak 2 (protocatechuic acid), among them, exhibited a steady peak shape, excellent peak form and separation, a moderate retention time, and no tailing phenomenon. Therefore, it was adopted as reference peak for fingerprint research. The RSD of the relative retention time of the common peaks of the fingerprints of 10 batches of *D. hancai* aqueous extract was calculated to be 0.05%∼0.30%; thus, it indicates that the chemical components of the 10 batches of samples were relatively stable and consistent. The relative peak area's RSD value, however, was 13.57%∼86.43% and fluctuated significantly, reflecting that the number of common components in *D. hancai* samples from various origins was quite different. The difference in content may be affected by climate, ecological environment, and various factors [21, 22], As the growth and quality of various traditional Chinese medicines are affected by ecological conditions, once the ecological environment changes, the content, color, and smell of traditional Chinese medicines will also change [23]. Concurrently, the harvest season and time of traditional Chinese medicine are also immensely close related to the quality of medicinal materials. Therefore, the content of active ingredients in the medicinal part of plants is different according to their growing stages [23, 24].

**Figure 1:**
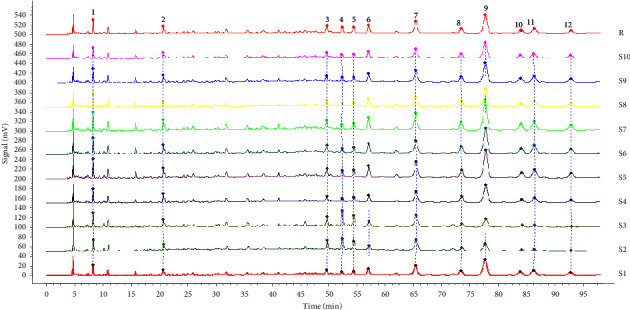
The overlay chromatograms of 10 batches of aqueous extract of *D. hancai*.

**Figure 2:**
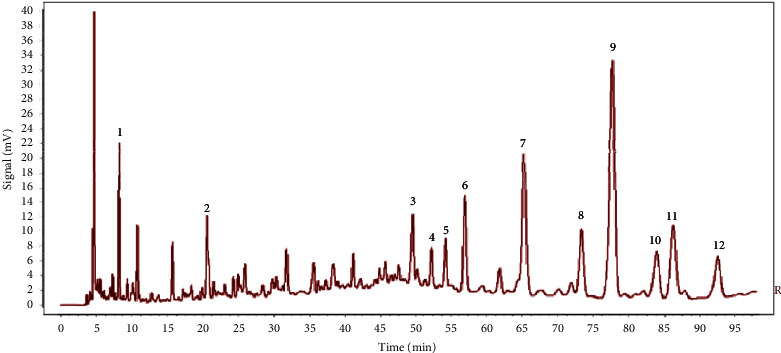
The reference fingerprint of 10 batches of aqueous extract of *D. hancai.*

**Figure 3:**
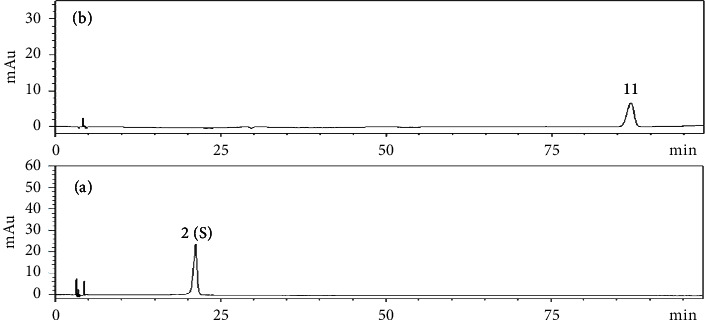
The HPLC chromatogram of reference solution. (a) 2(S): protocatechuic acid reference solution, (b) 11: vitexin reference solution.

The chromatograms of 10 batches of aqueous extract of *D. hancai* from different origins are present in Figure 1, the reference fingerprint in Figure 2, and the HPLC chromatogram of the reference solution in Figure 3. The similarity evaluation results are present in Table 2. The similarity of fingerprints of these batches of *D. hancai* aqueous extract was greater than 0.98, except for S2 and S3. It indicated that the samples of *D. hancai* aqueous were of a high degree of similarity and the overall quality of the samples was generally stable. The lower similarity of the fingerprints of S2 and S3 may be attributed to the differences in the contents of *D. hancai* herbs from these two producing areas due to different growing environment, climate, and seasonal factors [21–24]. In general, it conforms with the 0.9 fingerprint similarity criteria [25, 26].

#### 3.1.3. Cluster Analysis

**Figure 4:**
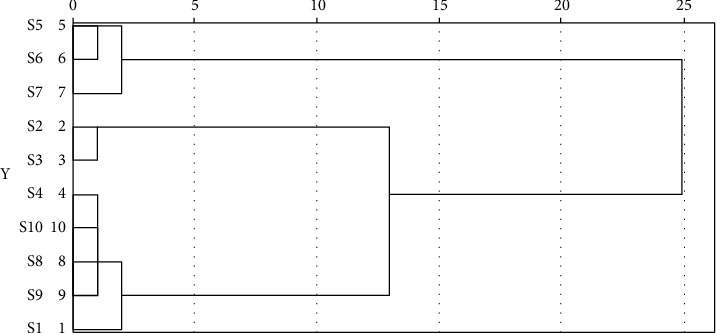
Cluster analysis results of 10 batches of aqueous extract samples of *D. hancai* from different producing areas.

The clustering analysis utilizes statistical methods to categorize unknown samples based on their variable characteristics in terms of similarity and is broadly applied in fingerprinting to classify different samples based on shared peaks, with the predominant clustering indicators being correlation coefficients and distances [27]. The results of the cluster analysis are displayed in Figure 4. In the figure, the *X*-axis represents the distance, while the *Y*-axis represents the spectrum number. The closer the distance is, the greater the similarity of the spectrum is, and the higher the reliability of the fingerprint spectrum is. The typical distances in the literature available are 5 [16], 10 [28], 15 [29], and so on. The results of the fingerprint clustering analysis in this experiment remained identical regardless of whether the distance selected was 5 or 10. Consequently, 10 batches of *D. hancai* aqueous extract samples from various producing regions can be categorized into 3 groups when the selection distance is 10, whereby cluster 1 is producing areas S5, S6, and S7; cluster 2 is producing areas S2 and S3; and cluster 3 is producing areas S1, S4, S8, S9, and S10. The 12 common peak areas of 10 batches of *D. hancai* from various origins functioned as the initial data for the cluster analysis, and the peak area represented the content of a chemical components. The results were grouped into three categories, demonstrating that samples from various production areas possessed varying contents. The difference in content between these 10 origins may be due to a variety of environmental conditions, which include local soil, the season, climate, the time of harvest, the development stage, and the level of maturity [21–24].

#### 3.1.4. Principal Component Analysis (PCA)

**Figure 5:**
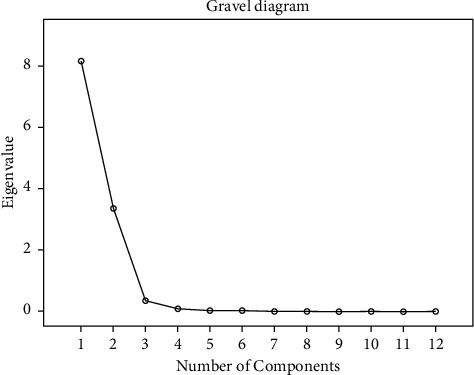
Gravel diagram for PCA of aqueous extract from *D. hancai*.

**Figure 6:**
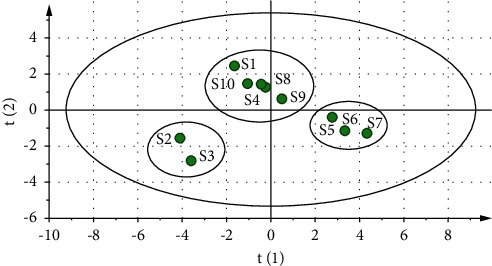
Principal component score map of 10 batches of aqueous extract of *D. hancai* from different origins.

The principal component analysis (PCA) method is widely employed in the research of traditional Chinese medicine. It attributes complex factors to several principal components, which are able to reduce data and simplify data dimensions [30]. The results of PCA of 10 batches of aqueous extract of *D. hancai* illustrated that two principal components were obtained with the characteristic value >1 as the selecting principle. The eigenvalue of principal component 1 is 8.160, the eigenvalue of principal component 2 is 3.355, and the variance contribution rates are 68.000% and 27.962%, respectively. The cumulative contribution rate is 95.962%, which can represent most of the chemical information of the samples. The results are presented in Table 3. At the same time, by observing the gravel diagram of the first two principal components (Figure 5), it can be observed that the first two principal components are steeper and the other components are relatively gentle. Thus, the first two principal component factors are selected for evaluation. Depending on the load's absolute value, the fundamental component's contribution may vary.  Table 4 demonstrates that the principal component 1 primarily represents information from the chromatographic peaks 1, 2, and 6–12, while the principal component 2 primarily reflects information from the chromatographic peaks 3, 4, and 5. According to the scoring diagram of PCA (Figure 6), the 10 batches of aqueous extract samples of *D. hancai* could be divided into 3 categories with S5, S6, and S7 were divided into one category, S2, and S3 were divided into another category, and S1, S4, S8, S9, and S10 were divided into the last category. This was consistent with the results of the cluster analysis.

### 3.2. Animal Experimental Results

In our previous experiment, the analgesic and anti-inflammatory effects of a high dose (57.6 g/kg), a middle dose (28.8 g/kg), and a low dose (7.2 g/kg) of aqueous extract of *D. hancai* collected from Nanning, Guangxi, were investigated. The results revealed that the high-dose group (57.6 g/kg) of aqueous extracts of *D. hancai* from different origins exhibited the most excellent analgesic and anti-inflammatory effects in mice. Therefore, the most effective dose of 57.6 g/kg crude drug was selected as the administration dose for mice. The results of the analgesic test demonstrated that the 10 batches of aqueous extract of *D. hancai* from different origins could effectively relieve the pain symptoms of mice induced by acetic acid, and its analgesic effect was superior as compared to that of the positive group. The results are presented in Table 5. The results of the anti-inflammatory experiment revealed that the 10 batches of aqueous extract of *D. hancai* from different origins could reduce the toe swelling degree of mice and inhibit the inflammation caused by carrageenan. The corresponding values are presented in Table 6.

### 3.3. Results of Spectral-Effect Relationship Analysis

The analysis methods of the spectrum-effect relationship of traditional Chinese medicine predominantly comprise gray relational analysis (GRA), bivariate correlation analysis (BCA), principal component analysis (PCA), partial least squares regression analysis (PLSR), multiple linear regression (MLR), canonical correlation analysis (CCA), artificial neural network (ANN) analysis, and so on [31]. GRA is employed to quantitatively describe the correlation between factors or things by correlation degree. Hence, it is able to reflect the contribution of fingerprint chromatographic peak components to drug efficacy thereby rendering it to be suitable for atlases with numerous factors and minimal information. At the same time, it is mainly able to analyze the correlation between each component and drug efficacy but is unable to effectively analyze the positive and negative effect trend of each component and drug efficacy [32]. The PLSR analysis can reflect the magnitude of the positive or negative contribution between each component and the drug effect. However, it is unable to effectively acquire the correlation between the component and the drug effect [33]. As a result, this method is employed to enable the two to complement each other and integrated applications, in order to be able to analyze the fingerprint and efficacy of the aqueous extract of *D. hancai* analgesic and anti-inflammatory spectrum-effect relationship more accurately.

#### 3.3.1. Results of GRA

The writhing inhibition rate and foot swelling inhibition rate of the 10 batches of *D. hancai* were utilized as the reference sequence, and the peak area of the common peaks of 10 batches of *D. hancai* was taken as the comparison sequence. The gray correlation degree is calculated after data averaging whereby the greater the correlation degree, the greater the contribution of the compounds that correspond to the peak and the efficacy of *D. hancai* [34].

The results of GRA are presented in Table 7. The correlation between the chromatographic peaks of *D. hancai* aqueous extract to the inhibition rate of analgesia in mice was from peak 1 > 8 > 2 > 9 > 7 > 10 > 5 > 11>4 > 6>12 > 3 in descending order, and the correlation between all peaks except peak 3 was larger than 0.7. The correlation between the chromatographic peaks of *D. hancai* aqueous extract and swelling inhibition rate in mice was from peak 1 > 8>2 > 9 > 7 > 5>10 > 11>4 > 6>12 > 3 in descending order, and the correlation between all peaks except peak 3 was larger than 0.7. The results indicated that the compounds represented by the peaks, except peak 3, were significantly correlated with the analgesic and inhibitory effects of *D. hancai*.

#### 3.3.2. Results of PLSR

The variable importance in projection (VIP) values is indicative of the magnitude of the role of each independent variable in the overall analysis system. Therefore, when the VIP value of the independent variable is >1.0, the independent variable is deemed to exert a significant contribution to the dependent variable [34].

**Figure 7:**
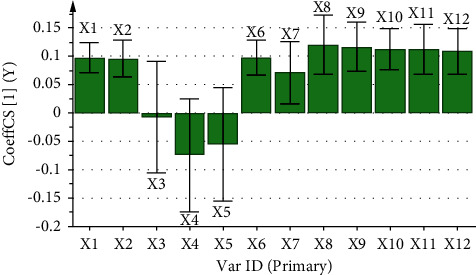
Partial regression coefficient diagram of analgesic spectrum-effect relationship of aqueous extract of *D. hancai*.

**Figure 8:**
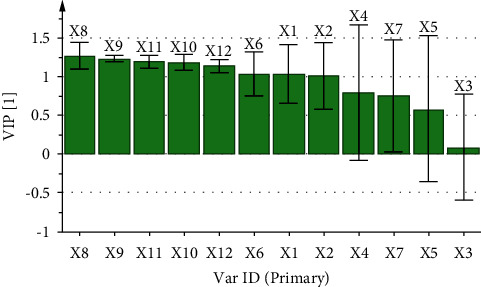
VIP diagram of common peak area and analgesic effect of aqueous extract of *D. hancai*.

The correlation coefficient and variable importance obtained by regression analysis of the fingerprint of aqueous extract of *D. hancai* and analgesic effect are presented in Table 8 and Figures 7 and 8. The results revealed that X1, X2, and X6∼X12 were all positively correlated with analgesic effect. The VIP values of X1, X2, X6, and X8∼X12 were all greater than 1, and the order was X8 > X9 > X11 > X10 > X12 > X6 > X1 > X2, indicating that the above chromatographic peaks exhibited had a significant effect on the analgesic effect of mice. When the content increased, the analgesic ability of the aqueous extract of *D. hancai* was significantly enhanced.

**Figure 9:**
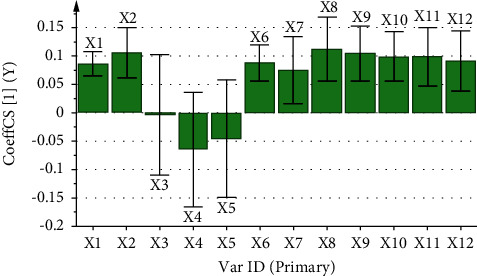
Partial regression coefficient diagram of anti-inflammatory spectrum-effect relationship of aqueous extract of *D. hancai*.

**Figure 10:**
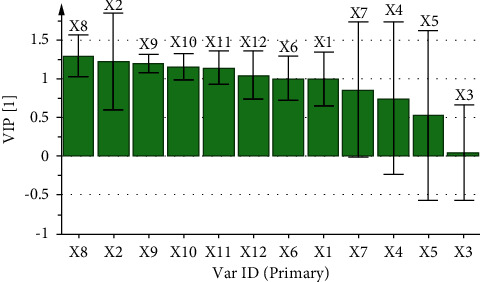
VIP diagram of common peak area and anti-inflammatory effect of aqueous extract of *D. hancai*.

The correlation coefficient and variable importance obtained by regression analysis of fingerprint and anti-inflammatory effect of aqueous extract of *D. hancai* are demonstrated in Table 9 and Figures 9 and 10. It can be observed that X1, X2, and X6∼X12 were positively correlated with the anti-inflammatory effect. The VIP values of X2, X6, and X8∼X12 were all greater than 1, and the order was X8 > X2 > X9 > X10 > X11 > X12 > X6. Hence, it indicated that the above chromatographic peaks exhibited a significant effect on the anti-inflammatory effect of mice.

#### 3.3.3. Comprehensive Analysis

The GRA and PLSR were combined to analyzed, and the gray correlation degree >0.7, the partial least squares regression coefficient was positive, and the VIP value was greater than 1 as the chromatographic peak screening conditions. Eventually, it was discovered that the main components of the analgesic effect of the aqueous extract of *D. hancai* were peaks 1, 2, 6, 8, 9, 10, 11, and 12; the key elements of the anti-inflammatory effect of the aqueous extract of *D. hancai* were peaks 2, 6, 8, 9, 10, 11, and 12.

## 4. Discussion

In the early stage of the experiment, the separation effects of different chromatographic columns (Thermo ODS-2 HYPERSIL, Agilent ZORBAX SB-Aq, Waters Atlantis C18) were comprehensively investigated. The baseline, resolution, signal intensity, and peak number of the three chromatograms were also thoroughly analyzed. The results revealed that the Waters Atlantis C18 column could effectively separate the main compounds with a more excellent peak shape. Simultaneously, it could also reflect more other chemical components in the aqueous extract of *D. hancai*. In addition, the different mobile phases (methanol-water, methanol-0.1% phosphoric acid, acetonitrile-water, acetonitrile-0.1% phosphoric acid), wavelength (220 nm, 240 nm, 260 nm, 280 nm, 300 nm, and 320 nm), flow rate (0.8 mL/min and 1 mL/min), column temperature (35°C and 40°C) were comprehensively investigated. Finally, by integrating with the peak signal intensity, peak number, resolution and baseline stability of the chromatogram, the most optimal chromatographic conditions for the fingerprint of the aqueous extract of *D. hancai* were successfully determined.

The correlation between HPLC fingerprint and analgesic and anti-inflammatory effects of aqueous extract of *D. hancai* from different origins was analyzed by GRA and PLSR. The GRA revealed that the correlation degree between each chromatographic peak of the aqueous extract of *D. hancai* and the inhibition rate of analgesia and toe swelling in mice were all >0.6, and the correlation degree of peaks 1, 2, 5, 7, 8, 9, and 10 were all >0.8, indicating that the chemical components represented by each chromatographic peak were correlated with the pharmacodynamic indexes, and the analgesic and anti-inflammatory effects of *D. hancai* might be the result of the combined action of multiple components. The PLSR revealed that peaks 1, 2, and 6∼12 were positively correlated with analgesic effect while peaks 2, 6, and 8∼12 were positively correlated with anti-inflammatory effect and that the VIP values were all greater than 1. Therefore, according to the results of GRA and PLSR, No. 1, 2, 6, and 8∼12 chromatographic peaks of the aqueous extract of *D. hancai* contributed significantly to the analgesic effect, which might serve as the material basis of the analgesic effect. Peaks 2, 6, and 8∼12 of *D. hancai* aqueous extract exhibited a higher degree of contribution to the anti-inflammatory effect, possibly accounting for the material basis of the anti-inflammatory effect of the *D. hancai* aqueous extract. Protocatechuic acid is at peak 2 and vitexin is at peak 11, among others. It was discovered that protocatechuic acid [35, 36] and vitexin [37, 38] both exhibited excellent analgesic effects and obvious anti-inflammatory effects, which could inhibit the production of inflammatory mediators and inflammatory factors as well as slow down the inflammatory response. It can be observed that protocatechuic acid and vitexin may be one of the potential analgesic and anti-inflammatory active ingredients of *D. hancai*. However, the remaining characteristic peaks have not been successfully identified. Therefore, in the follow-up study, the structure of unknown chromatographic peaks should be identified by Q-TOF or LC-MS/MS to further clarify the active components related to analgesic and anti-inflammation in the aqueous extract of *D. hancai*.

## 5. Conclusions

In this research, 12 similar peaks and the HPLC fingerprints of 10 batches of *D. hancai* aqueous extract were successfully established. Peaks 2 and 11 were identified as protocatechuic acid and vitexin, respectively, through comparison to reference chemicals. The evaluation of fingerprint similarity yielded data that indicated that exception of S2 and S3, all other producing areas' fingerprint similarity was larger than 0.98. The results of cluster analysis and PCA were consistent, and 10 batches of medicinal materials were clustered into 3 categories, indicating that the method can be adopted for the quality evaluation of the aqueous extract of *D. hancai*.

In this experiment, based on the quantitative analysis of HPLC fingerprint characteristic peaks as well as the analgesic and anti-inflammatory data of aqueous extract of *D. hancai* from 10 different origins, the correlation between the analgesic and anti-inflammatory spectrum of *D. hancai* was obtained by GRA and PLSR. The results revealed that peaks 1, 2, 6, 8, 9, 10, 11, and 12 in the aqueous extract of *D. hancai* were the chromatographic peaks that contributed significantly to the analgesic effect, and peaks 2, 6, 8, 9, 10, 11, and 12 were the chromatographic peaks that contributed substantially to the anti-inflammatory effect. The compounds may be the material basis for the analgesic and anti-inflammatory effects of the aqueous extract of *D. hancai.* Therefore, this experiment is able to serve as the basis for further in-depth exploration of the analgesic and anti-inflammatory substances and quality evaluation of *D. hancai.*

## Figures and Tables

**Table 1:** Source information table of *D. hancai*

Numbers	Producing areas	Purchasing period
S1	Nanning, Guangxi	2019.03
S2	Yulin, Guangxi	2019.03
S3	Liuzhou, Guangxi	2019.04
S4	Laibin, Guangxi	2019.04
S5	Qinzhou, Guangxi	2019.05
S6	Guilin, Guangxi	2019.05
S7	Hezhou, Guangxi	2019.05
S8	Baise, Guangxi	2019.06
S9	Hechi, Guangxi	2019.06
S10	Wuzhou, Guangxi	2019.06

**Table 2:** 10 batches of aqueous extract from *D. hancai* similarity evaluation results.

Numbers	Similarity
S1	0.990
S2	0.857
S3	0.850
S4	0.991
S5	0.992
S6	0.988
S7	0.991
S8	0.986
S9	0.994
S10	0.992

**Table 3:** Principal component eigenvalue and variance contribution rate of aqueous extract of *D. hancai*.

Principal components	Eigenvalue	Variance contribution rate (%)	Cumulative contribution rate (%)
1	8.160	68.000	68.000
2	3.355	27.962	95.962
3	0.348	2.897	98.859
4	0.090	0.754	99.613
5	0.024	0.202	99.815
6	0.017	0.141	99.956
7	0.004	0.034	99.990
8	0.001	0.009	99.999
9	0.000	0.001	100.000

**Table 4:** Principal component load matrix of aqueous extract from *D. hancai*.

Peaks	Principal component	
	1	2
1	0.888	0.366
2	0.877	0.273
3	0.262	0.933
4	−0.386	0.917
5	−0.202	0.977
6	0.968	0.148
7	0.826	0.543
8	0.958	−0.227
9	0.987	−0.158
10	0.991	−0.083
11	0.971	−0.206
12	0.954	−0.201

**Table 5:** Experimental results of writhing induced by acetic acid in mice.

Groups	Dosage of extract (g/kg) (crude drug 57.60 g/kg)	Twisting number (time) ($\bar{x}$ ± *s*)	Analgesic inhibition ratio (%)
Positive group	—	28.5 ± 4.2	—
Rotundine group	0.03	20.3 ± 3.9^*∗∗*^	28.77
Nanning, Guangxi	6.07	12.9 ± 4.3^*∗∗*^	54.74
Yulin, Guangxi	3.61	17.3 ± 4.2^*∗∗*^	39.30
Liuzhou, Guangxi	4.20	17.1 ± 3.8^*∗∗*^	40.00
Laibin, Guangxi	5.47	13.2 ± 4.3^*∗∗*^	53.68
Qinzhou, Guangxi	6.47	11.1 ± 4.0^*∗∗*^	61.05
Guilin, Guangxi	7.42	11.3 ± 4.1^*∗∗*^	60.35
Hezhou, Guangxi	4.63	10.4 ± 3.8^*∗∗*^	63.51
Baise, Guangxi	4.17	13.5 ± 3.6^*∗∗*^	52.98
Hechi, Guangxi	5.30	14.2 ± 4.2^*∗∗*^	50.18
Wuzhou, Guangxi	5.17	15.6 ± 4.2^*∗∗*^	44.91

*Note.* Compared with the model group: ^*∗*^*P*  < 0.05; ^*∗∗*^*P* < 0.01.

**Table 6:** Experimental results of toe swelling induced by carrageenan in mice.

Groups	Dosage of extract (g/kg) (crude drug 57.60 g/kg)	Swelling degree (mg) ($\bar{x}$ ± *s*)	Swelling inhibition rate (%)
Model group	—	47.61 ± 9.99	—
Positive group	0.006	24.03 ± 8.16^*∗∗*^	49.52
Nanning, Guangxi	6.07	29.95 ± 6.42^*∗∗*^	37.10
Yulin, Guangxi	3.61	33.30 ± 9.90^*∗∗*^	30.06
Liuzhou, Guangxi	4.20	32.46 ± 8.21^*∗∗*^	31.82
Laibin, Guangxi	5.47	26.03 ± 7.71^*∗∗*^	45.34
Qinzhou, Guangxi	6.47	27.92 ± 7.87^*∗∗*^	41.36
Guilin, Guangxi	7.42	27.19 ± 7.77^*∗∗*^	42.90
Hezhou, Guangxi	4.63	25.48 ± 6.87^*∗∗*^	46.50
Baise, Guangxi	4.17	31.41 ± 9.90^*∗∗*^	34.02
Hechi, Guangxi	5.30	28.10 ± 10.65^*∗∗*^	40.98
Wuzhou, Guangxi	5.17	30.94 ± 9.70^*∗∗*^	35.02

*Note.* Compared with the model group: ^*∗*^*P*  < 0.05; ^*∗∗*^*P* < 0.01.

**Table 7:** GRA results of 12 common peaks in *D. hancai* cochinchinensis and analgesic and anti-inflammatory effects.

Common peak number	Analgesic effect		Anti-inflammatory effect	
	Correlation	Correlation order	Correlation	Correlation order
1	0.9304	1	0.9415	1
2	0.8940	3	0.9238	3
3	0.6853	12	0.6973	12
4	0.7660	9	0.7876	9
5	0.8035	7	0.8240	6
6	0.7362	10	0.7527	10
7	0.8138	5	0.8344	5
8	0.9244	2	0.9357	2
9	0.8591	4	0.8691	4
10	0.8070	6	0.8142	7
11	0.7922	8	0.7999	8
12	0.7297	11	0.7349	11

**Table 8:** Partial regression coefficient results of characteristic peak area of fingerprint of aqueous extract of *D. hancai* and analgesic inhibition rate in mice.

Characteristic peak	Partial regression coefficient
X1	0.0975
X2	0.0959
X3	−0.0082
X4	−0.0749
X5	−0.0551
X6	0.0979
X7	0.0709
X8	0.1194
X9	0.1165
X10	0.1119
X11	0.1127
X12	0.1077

**Table 9:** Partial regression coefficient results of characteristic peak area of fingerprint of aqueous extract of *D. hancai* and inhibition rate of toe swelling in mice.

Characteristic peak	Partial regression coefficient
X1	0.0862
X2	0.1058
X3	−0.0042
X4	−0.0646
X5	−0.0459
X6	0.0873
X7	0.0742
X8	0.1125
X9	0.1040
X10	0.0998
X11	0.0988
X12	0.0910

## Data Availability

The data used to support the findings of this study are included within the article.
